# The role of parental child marriage in children's food security and nutritional status: a prospective cohort study in Indonesia

**DOI:** 10.3389/fpubh.2024.1469483

**Published:** 2024-12-10

**Authors:** Isnawati Hidayah, Asep Suryahadi, Flaviana Palmisano, Jessica C. Kiefte-de Jong

**Affiliations:** ^1^Department of Public Health and Primary Care/Health Campus The Hague, Leiden University Medical Center, Leiden, Netherlands; ^2^Department of Economics and Law, Sapienza University of Rome, Rome, Italy; ^3^SMERU Research Institute, Jakarta, Indonesia

**Keywords:** child marriage, food insecurity, stunting, children, IFLS, intergenerational, Indonesia

## Abstract

**Objectives:**

Assessing children's food and nutrition security in Indonesia, especially among children from parents who experienced child marriage, is crucial for policymakers. This study investigates the role of parental child marriage in children's food security and nutritional status.

**Methods:**

We analyze data from Indonesia Family Life Survey (IFLS) wave 4 (2007) and 5 (2014), involving 1,612 households. We employ OLS and binary logit regression analysis.

**Results:**

Our analysis reveals that parental child marriage is associated with higher probability of children being stunted and experiencing food insecurity. Additionally, parental child marriage correlates with higher BMI-for-age *z*-scores, which increase the risk of obesity, and lower Food Consumption Scores (FCS).

**Recommendation:**

Implementing community initiatives, economic empowerment, healthcare access, and gender-sensitive and integrated policies is crucial for enhancing food security and improving nutritional status among childen in families affected by child marriage.

## 1 Introduction

One enduring issue in Indonesia is chronic malnutrition and food insecurity experienced by households where parents entered into child marriage. As per the Convention on the Rights of the Child (CRC), child marriage is defined as the union of individuals under the age of 18 (1). In addition, child marriage has a prominent place in the Sustainable Development Goals (SDGs), which has placed emphasis on gender equality and empowerment of women and girls. Target 5.3 of the SDGs specifically aims to eradicate all harmful practices, including child, early, and forced marriage, as well as female genital mutilation, by the year 2030.

Unfortunately, (2) reveales that Indonesia has one of the highest numbers of child marriages in the world, indicating that individuals marry before reaching 18 years of age. The prevalence of child marriage in 20 out of 38 provinces remains above the national average (3). To overcome the problem, the Indonesian parliament has unanimously passed a law to increase the minimum legal age of marriage for women from 16 to 19 years, aligning it with the existing legal age requirement for men (House of Representatives of the Republic of Indonesia, 2019). This marked a significant breakthrough for Indonesia in its efforts to combat child marriage. Indonesia's National Mid-term Development Plan (2020–2024) targets to reduce child marriage rates, supported by the development of a National Strategy on the Prevention of Child Marriage with defined goals (4).

**Figure 1:**
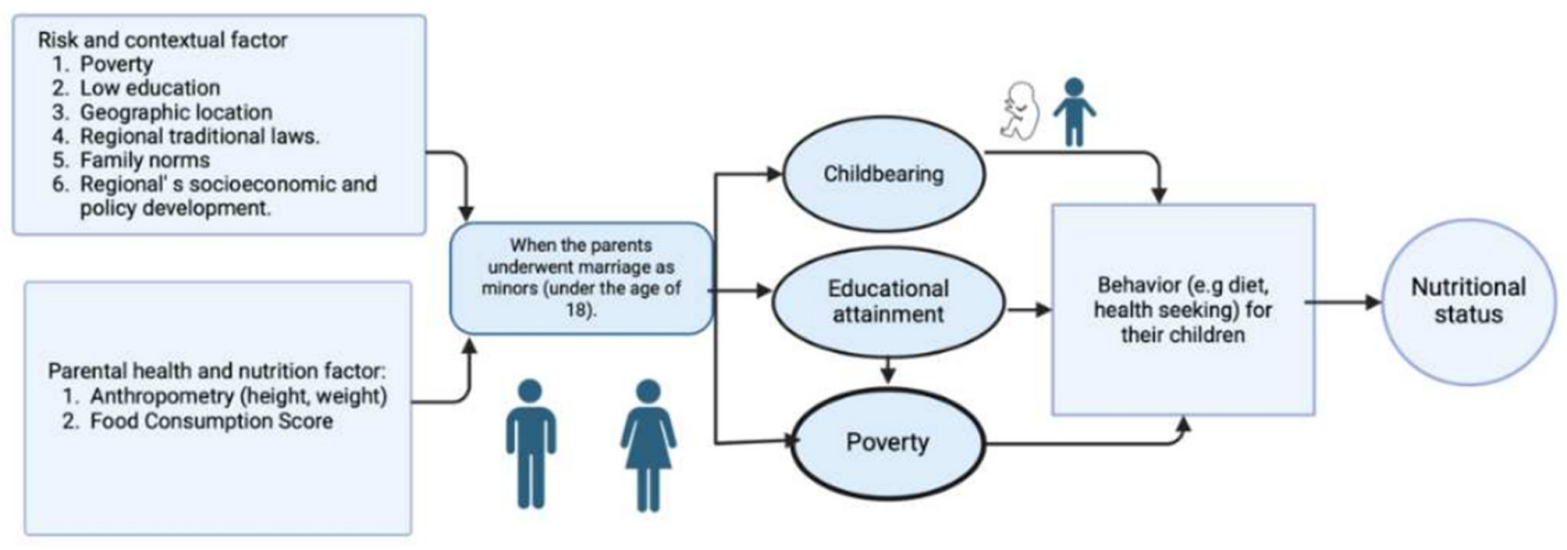
Conceptual model of how girl child marriage can impact health and nutritional status.

Several factors serve as driving forces for child marriage (see Figure 1), including poverty, low education levels (5), and difficult geographic location. The findings from prior studies highlight that poverty (6), limited education, large family sizes, societal pressures, and familial responsibilities (7) are key factors leading to child marriage. The literature also highlights the lack of opportunities for continued education and paid employment as factors driving child marriage (8), contributing to poor economic outcomes and poverty among women (9). Indonesian women who marry before the age of 19 tend to experience a lower quality of life for themselves and their children compared to those who delay marriage for several years (10). Child marriage significantly compromises the food security of children by limiting access to education and healthcare, perpetuating poverty, reducing security and disrupting household dynamics (see Figure 1).^1^

In terms of food security, Indonesia was ranked 70th out of 117 nations in 2019 (12). According to the ADB (13), ~8.3% (20.7 million people) of Indonesians were at risk of hunger. In rural areas, poverty is the main factor driving households into food insecurity (14). According to Statistics Indonesia (BPS), in 2018, Indonesia's rural poverty reached 13.2% of the total population. Besides, 14.5% of Indonesian rural districts suffer from serious malnutrition and food insecurity (15).

In general, child marriage can either be a driving force (16–18) or a consequence of food insecurity and malnutrition. However, there are relatively few studies that focus on its consequences, particularly in the context of Indonesia. Child marriage, poverty, and food insecurity are unquestionably interconnected, with consequences that can persist across generations. This is consistent with the findings from (19), which highlight that poverty has become a major driver of child marriage. In a study from (20), it was found that in Indonesia, parents living in poverty often marry off their daughters to alleviate the financial strain of raising them. As a result, parents are more likely to seize any opportunity for their daughters to marry (20). Moreover, parents who have experienced child marriage during their early years potentially face challenges in accessing decent employment, adequate healthcare and food insecurity (9).

The occurrence of child marriage is notably higher among both females and males in rural regions (37% female, 12% male) compared to urban areas (4). In addition, there are other important determinants of child marriage, such as gender, ethnicity, and religion, which are relevant in the Indonesian context.

In order to solve malnutrition and food insecurity, the Indonesian government has created regulations, in particular by passing law No. 18/2012 on Food, which stipulates that everyone has the right to access all their basic needs, such as sufficient food in both quantity and quality, to enable them to have food sovereignty and security. Unfortunately, the regulations have failed to solve malnutrition and food security for various reasons, including overly optimistic expectations, inadequate collaboration with other stakeholders, poor implementation, overly complex bureaucracy, and corruption (21).

As outlined above, issues of food and nutrition security in Indonesia can be seen as a consequence of child marriage. However, research addressing the intergenerational consequences of child marriage is still limited in Indonesia. Especially, the impact on children's nutritional status and food insecurity in the aftermath of child marriage has not been thoroughly explored. Hence, assessing intergenerational food and nutrition security in Indonesia, especially from parents that experienced child marriage, is crucial for policymakers in their efforts to resolve the issue. In addition, religion and ethnicity are crucial to explore in this study, as different religious and ethnic backgrounds, along with gender, influence child marriage practices, which may impact food security and nutritional status. Lastly, child marriage is also influenced by social norms prevalent in society (22).

Hence, the objective of this study is to examine the association between parents who experienced child marriage and the food security status of their children across different socioeconomic strata in Indonesia, to investigate whether food and nutrition security challenges persist across generations. Furthermore, to check for heterogeneity in the associations, this study also explores the associations within different ethnic groups and religions.

The rest of the paper is structured as follows: Section 1 presents an introduction that also describes the child marriage situation in Indonesia. Section 2 presents the data and methods used for the analysis. Section 3 provides the results, that then discussed in Section 4. Section 5 concludes.

## 2 Materials and methods

### 2.1 Data sources

Data for this study are sourced from the Indonesia Family Life Survey (IFLS), an ongoing longitudinal study initiated in 1993. The IFLS underwent rigorous institutional review board (IRB) scrutiny and received approval from the RAND Corporation and relevant institutions in Indonesia. These entities conducted thorough and appropriate reviews of the study's human subject considerations (23).

The study population encompass women and men aged 21—85 years in 2007 who had experienced child marriage, along with their children aged 0–15 in 2014. We construct a parents dataset using IFLS 4 (2007) from 13,535 households, supplemented by information on these parents' socioeconomic status seven years later using IFLS 5 (2014). Additionally, the children's dataset is derived from IFLS 5 (2014), which covers 16,204 households. The dataset encompasses a total of 1,612 households, representing various household compositions. Of these, 1,326 households consist of married couples, some of whom have additional wives. Additionally, the dataset includes 235 households of widowed females and 51 households of widowed males. In total, there are 1,635 sons and 1,647 daughters living in these households. Both parents and children reside in the same households. These diverse household compositions offer a comprehensive view of household structures in Indonesia.

### 2.2 Indicator

#### 2.2.1 Food security indicator

As a measure of food security, we use the Food Consumption Score (FCS) (24–26). This indicator is a combined score derived from the variety of foods consumed by households, the frequency of their food consumption, and the nutritional value of different food groups.^2^ We aggregate FCS into three categories of Food Consumption Group (FCG): (1) “poor” if FCS falls below 21, (2) “borderline” if FCS ranges from 21 to 35, and (3) “acceptable” if FCS is above 35 (25, 26). The Food Security indicator (FS) is then obtained by further partitioning the FCG into two categories: (1) “food insecure” if the FCG is categorized in the borderline or poor groups and (2) “food secure” if the FCG is categorized in the acceptable group (25, 26). The dependent variables encompasses Child's FCS and FS status *(yt)*, while the control variables include parent's FCS and FS status *(xt)*.

#### 2.2.2 Nutritional status

The Body Mass Index (BMI), also known as the Quetelet Index, serves as a vital tool for assessing nutritional status among adults aged 15 and above. Calculated as weight in kilograms divided by height in meters squared (kg/m^2^), BMI offers a straightforward measure of an individual's weight relative to their height. This index holds significant importance in gauging nutritional wellbeing. Indonesian Ministry of Health (27) outlined that individuals with a BMI exceeding 24.9 are categorized as overweight or obese, posing significant challenges to public health.

Nutritional status for children aged 0–15 years is determined using the 2007 WHO references (28). This involves calculating *Z*-scores, a method that expresses anthropometric measurements as deviations from the mean reference value in terms of standard deviations. *Z*-scores are commonly computed for weight-for-height, weight-for-age, height-for-age, and BMI-for-age measurements. According to World Health Organization (28), percentiles are derived within the range of *z*-scores between -3 and 3 to ensure consistency, as percentiles beyond ±3 SD remain unchanged with variations in equivalent *z*-scores. In this study, we highlight the use of height-for-age growth indicator. This indicator is crucial for assessing the growth and nutritional status of children. The height-for-age indicator measures whether a child's height is appropriate for their age and sex compared to a reference population of healthy children.

Based on the height-for-age *Z*-score (HAZ), children fall into several categories: those with a HAZ between -2 and +2 are considered to have normal growth; those with a HAZ between -2 and -3 are classified as moderately stunted, indicating chronic malnutrition or long-term health issues; those with a HAZ below -3 are considered severely stunted, reflecting severe chronic malnutrition or significant health problems; and those with a HAZ above +2 are noted as tall-for-age, indicating excessive growth. The HAZ is calculated by comparing a child's height to the median height of a reference population of the same age and sex, expressed in standard deviations from the reference median.

In this study, the dependent variables encompass the child's z-score using BMI-for-age and the child's malnutrition status using height-for-age (*yt*) (likelihood of stunting or not). Meanwhile, the control variables include parent's BMI (*xt*). For interpretation, we follow the guidelines outlined by (28).^3^

#### 2.2.3 Child marriage

Child marriage pertains to a legal or informal partnership in which either one or both individuals involved are below the age of 18. In this study, a binary indicator is used, denoted as 1 if a parent experienced child marriage, and 0 otherwise. About 97.94% of child marriage occur during their first marriage. We include in the data only if the parents are still in a marital or cohabiting relationship or one of their partner(s) has passed away already. The child marriage status serves as the main independent variable *(xt)* in this study.

**Figure 2:**
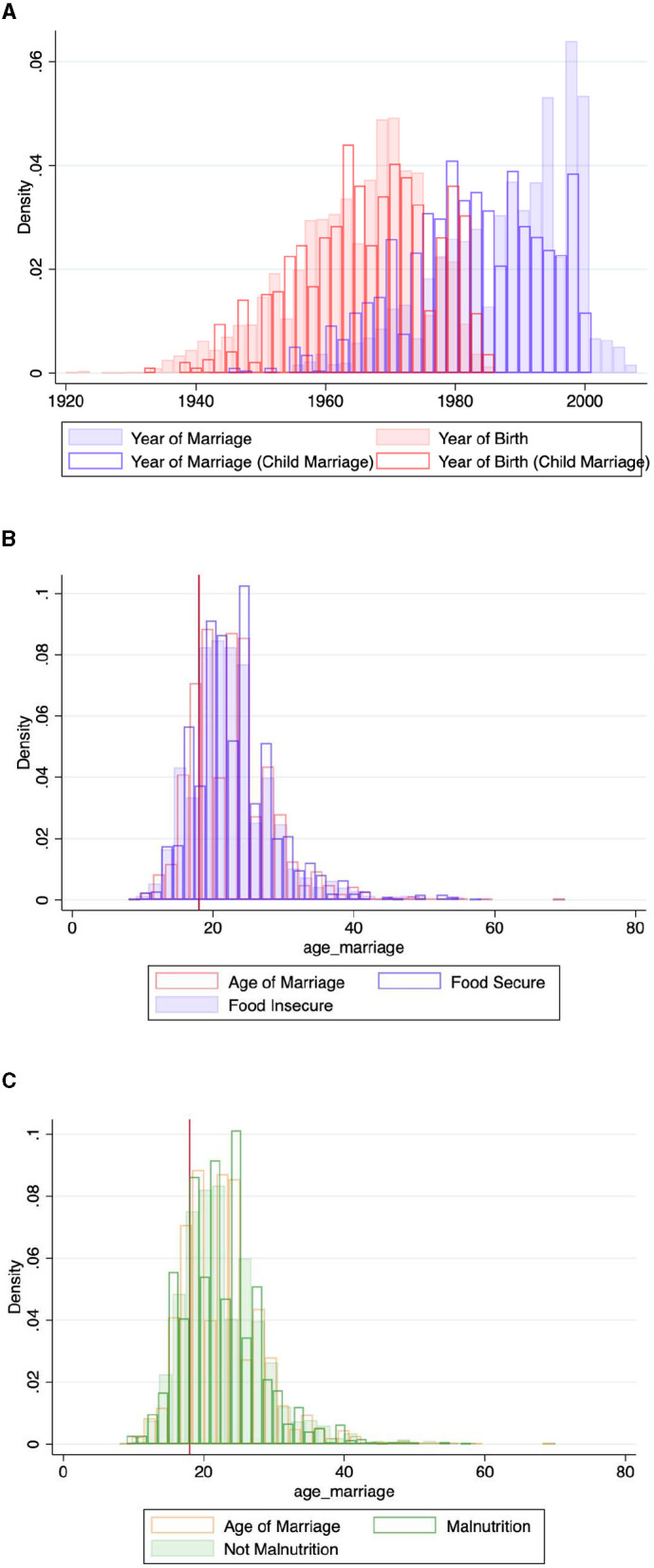
Distribution on year of marriage, year of birth, food and nutrition security on parents. **(A)** Distribution on year of marriage and year of birth, the red line marks the benchmark age of 18 years for marriage. **(B)** Food security and age of marriage, the red line marks the benchmark age of 18 years for marriage. **(C)** Malnutrition and age of marriage, the red line marks the benchmark age of 18 years for marriage.

Figure 2 illustrates the distribution of the year of marriage compared to the year of birth, providing insight into the distribution of parental child marriage information.

#### 2.2.4 Control variables

To address potential confounding factors, we evaluated several key variables,^4^ encompassing individual factors, household characteristics, community dynamics, and regional indicators. For comprehensive details regarding the control variables, namely: children's food security status; the interaction between parental child marriage status and food security status in 2007; child gender and age; child marriage status; parental food security status in 2007 and 2014; employment status; household size; religious affiliation (Islam, yes/no); monthly household expenditure and food expenditure share; age of household members; per capita monthly expenditure in 2007 (PPP); rural residency (yes/no); gender of key household members (female, yes/no); marital status (married/cohabiting); midwifery services availability in villages; presence of ASKESKIN (health insurance) and RASKIN (subsidized rice programs) in villages; tracking rural residency in 2014 (yes/no); per capita monthly expenditure in 2014; and enforcement of traditional laws and changes in marriage laws, expenditure allocation across various food items such as staples and dairy products, and non-food items such as alcohol, education, medical expenses, regional GDP and Gini coefficient.

### 2.3 Statistical analysis

In this analysis, we utilize two regression models: Ordinary Least Squares (OLS) and binary logit regression. The OLS regression assesses children's FCS and BMI-for-age z-score as the dependent variables. Meanwhile, the binary logit regression examines child food security and malnutrition status (specifically, the likelihood of being stunted or not) as distinct dependent variables in separate analyzes.

For OLS regression, considering a linear regression model:


$$y_{i}=\beta_{0}+\beta_{1}x_{i1}+\beta_{2}x_{i2}+\ldots +\beta_{k}x_{ik}+\epsilon_{i}$$

For binary outcome data, the dependent variable *y* takes one of two values, The logit model is the mathematical form (29):


$$p_{i}=\mathrm{Pr}\left[y_{i}=1|x\right]=\frac{\exp\left(\beta_{1}+\beta_{2}x\right)}{1+\exp\left(\beta_{1}+\beta_{2}x\right)}$$

with β is the parameter and ensures that 0 < *p*_*i*_ < 1.

### 2.4 Model selection and robustness check

For robustness check, to compare alternative models, the likelihood criteria and the Bayesian Information Criteria (BIC), the Akaike Information Criteria (AIC) are used, following Cameron (29) and Hardin and Hilbe (30), to check whether the log model is not different from the non-log model. AIC and BIC indicate that a model with the smaller value of the information criterion is considered better.

Our study includes several model specifications, utilizing full control variables encompassing individual characteristics (personal expenditure, religion, ethnicity, education), household factors (expenditure on various food sources and other expenses), community variables (local marriage laws, government policies on food and poverty alleviation in village level), and regional indicators (regional GDP and Gini coefficient). We also considered reduced control variables derived from this comprehensive set. Moreover, robust standard errors are computed to address issues such as heteroscedasticity or other model misspecifications.

In examining the relationship between child marriage and nutritional status or z-score, we compared results using various growth indicators (weight-for-height, weight-for-age, height-for-age, and BMI-for-age). To ensure robustness by employing both robust standard errors and standard errors clustered at the Enumeration Area (EA), reflecting community-level effects were used. Our analyzes consistently yield robust results, providing confidence in the reliability of our findings. Moreover, observations in our dataset were weighted to reflect their significance in representing the target population.

## 3 Results

### 3.1 Sample characteristics

The Table 1 provides detailed statistics on various variables concerning demographic and social characteristics at both the individual and household levels, community and regional economic data. Regarding religion, 92.17% identify as Islam, 0.79% as Protestant, 1.07% as Catholic, and 5.97% as Hindu. In terms of current activity, 65.57% of individuals are employed or contributing to income generation, 0.24% are actively seeking employment, 29.95% are housekeepers, 1.71% are retired, and 2.16% are unemployed.

**Table 1:** Descriptive statistics.

	**2007**		**2014**	
**Variable**	**Obs or N**	**Mean (std. dev) or %**	**Obs or N**	**Mean (std. dev) or %**
**Parent**				
Log of household expenditure in 2007	3,251	14.206 (0.609)		
Regional gini index 2014			3,282	0.3515(0.0132)
Regional gini index 2007	3,282	0.3993 (0.0275)		
Log of regional GDP 2014			3,282	12.922 (1.17)
Food expenditure share in 2007	3,251	57.65 (14.947)		
Non-food expenditure share in 2007	3,251	42.35 (14.947)		
**Parent's religion**				
Islam	3,025	92.17		
Protestant	26	0.79		
Catholic	35	1.07		
Hinduism	196	5.97		
**Parent's latest activity**				
Work/helping to get income	2,152	65.57		
Looking for work	8	0.24		
Housekeeper	983	29.95		
Retired	56	1.71		
Unemployment	71	2.16		
**Sex of parent**				
Male	1,377	41.96		
Female	1,905	58.04		
**Ethnicity of parent**				
Javanese	1,383	42.14		
Sundanese	465	14.17		
Bali	205	6.25		
Bugis	95	2.89		
Maduranese	197	6.00		
Sasak	312	9.51		
Banjar	172	5.24		
Bima-Dompu	144	4.39		
Cirebon	137	4.17		
**Parent's education per 2007**				
Complete elementary	911	27.76		
Complete junior high	347	10.57		
Complete senior high	553	16.85		
Incomplete elementary	911	27.76		
Incomplete junior high	112	3.41		
Incomplete senior high	65	1.98		
No education	383	11.67		
**Parent's experience on child marriage**				
No	2,224	67.76		
1	1,058	32.24		
**Children** **Sex of child**				
Boy			1,635	49.82
Girl			1,647	50.18
**Age**			3,282	7.850396 (4.310048)
**Child's malnutrition status (height-for-age)**				
Normal			2,385	72.67
See note 1			4	0.12
Severely stunted			197	6.00
Stunted			663	20.20
**Child's FCS category**				
Poor	51	1.55		
Borderline	281	8.56		
Acceptable	2950	89.88		

Regarding children's gender, 49.82% are boys and 50.18% are girls. Among parents, 41.96% are male and 58.04% are female. In terms of education, 27.76% have completed elementary school, while 11.67% have no formal education. Lastly, regarding child marriage that occurred on or before 2007, 67.76% of individuals did not experience child marriage, while 32.24% did.

### 3.2 Parental child marriage and children's nutritional status

In this section we compared the results obtained using OLS (see Table 2) and binary logistic regression (see Table 3), with *z*-score (children) and malnutrition status (children) as the dependent variables, aiming to provide a more comprehensive analysis.

**Table 2:** Parental child marriage and children's *z*-score—ordinary least squares.

	**Model 1**	**Model 2**	**Model 3**	**Model 4**	**Model 5**
**Variables**	**Children's** ***z*****-score**	**Children's** ***z*****-score**	**Children's** ***z*****-score**	**Children's** ***z*****-score**	**Children's** ***z*****-score**
Parent's child marriage experience	0.102^**^	0.164^**^	0.185^**^	0.126^*^	0.202^**^
	(0.0470)	(0.0711)	(0.0931)	(0.0657)	(0.0939)
Interaction parental child marriage status* parental food					
Security status in 2007		-0.0927	-0.152		-0.113
		(0.0786)	(0.112)		(0.0971)
Children's FCS		-0.00108	0.000214	-0.00143	-0.00138
		(0.00118)	(0.00173)	(0.00146)	(0.00146)
Children food security status			-0.149		
			(0.0966)		
Parent's FCS in 2014			-0.00161	-0.00107	-0.00108
			(0.00151)	(0.00155)	(0.00155)
Parent's FCS in 2007			0.00124	0.000836	0.00180
			(0.00285)	(0.00226)	(0.00239)
Parent's BMI in 2014			0.0101**	0.0158***	0.0153**
			(0.00503)	(0.00611)	(0.00618)
Parent's BMI in 2007			0.000154	0.000287**	0.000280**
			(0.000164)	(0.000134)	(0.000136)
Socioeconomics characteristics	No	No	No	Yes	Yes
**Interaction child marriage and parent**					
Food security in 2007	No	Yes	Yes	No	Yes
Constant	6.827^***^	6.885^***^	6.706^***^	6.278^***^	6.253^***^
	(0.0299)	(0.0805)	(0.163)	(1.303)	(1.309)
Observations	3,029	3,029	2,598	2,255	2,255
R-squared	0.003	0.004	0.009	0.044	0.045

Standard errors in parentheses.

* *p* < 0.05, ** *p* < 0.01, *** *p* < 0.001.

Each observation in the dataset is weighted according to its significance in representing the target population. Robust standard errors are computed to account for heteroscedasticity or other forms of model misspecification. Models 4 and 5 incorporate socio-economic characteristics at various levels: individual (personal expenditure, religion, ethnicity, education), household (expenditure shares for different food sources), community (local laws on marriage, government policies), and regional (regional GDP and Gini coefficient). Model 4 does not include an interaction variable, whereas Model 5 does.

**Table 3:** Parental child marriage and children's likelihood of being stunted (malnutrition status)—binary logit regression [odd ratio].

	**Model 1**	**Model 2**	**Model 3**	**Model 4**	**Model 5**
**Variables**	**Malnutrition status**	**Malnutrition status**	**Malnutrition status**	**Malnutrition status**	**Malnutrition status**
Parent's child marriage status (Yes/No)	1.409^***^	1.511^**^	1.590^**^	1.502^*^	1.377^**^
	(0.164)	(0.273)	(0.325)	(0.327)	(0.208)
Interaction child marriage and parent food security in 2007		0.886	0.931	0.881	
		(0.179)	(0.220)	(0.216)	
Child's FCS		1.001	1.000	1.002	1.002
		(0.00282)	(0.00323)	(0.00400)	(0.00399)
Child's food security status				0.959	0.965
				(0.244)	(0.245)
Parent' FCS in 2014			1.013^***^	1.014^***^	1.014^***^
			(0.00368)	(0.00379)	(0.00379)
Parent's FCS in 2007			0.989^**^	1.002	1.000
			(0.00528)	(0.00589)	(0.00530)
Parent's BMI in 2014			0.957^***^	0.974^*^	0.974^*^
			(0.0131)	(0.0147)	(0.0147)
Parent's BMI in 2007			1.001	1.000	1.000
Socioeconomics characteristics	No	No	No	Yes	Yes
Interaction child marriage and parent food security in 2007	No	Yes	Yes	No	Yes
Constant	0.300^***^	0.280^***^	0.604	0.418	0.443
	(0.0208)	(0.0519)	(0.247)	(0.739)	(0.778)
Observations	3,029	3,012	2,581	2,557	2,557

Standard errors in parentheses.

* *p* < 0.05, ** *p* < 0.01, *** *p* < 0.001.

Each observation in the dataset is weighted according to its significance in representing the target population. Additionally, it indicates that robust standard errors should be computed to account for heteroscedasticity or other forms of model misspecification. Model 4 and Model 5 incorporate socio-economic characteristics at various levels: individual (personal expenditure consumption, religion, ethnicity, education), household (household expenditure, expenditure shares for different food sources, and other factors), community (local laws on marriage, government policies on food and poverty eradication), and regional (regional GDP and Gini coefficient). The distinction between the two models is that Model 5 does not include an interaction variable, whereas Model 4 does.

The Table 2^5^ presents the results from four models analyzing the determinants of children's *z*-scores using OLS. Each model included different sets of control variables.^6^

The key variable of interest, parent's child marriage experience (yes/no), showed a positive and statistically significant association with children's *z*-scores across all models (see Table 2). To be more precise, in Model 1, the coefficient is 0.102(*p* < 0.05), indicating that having a parent who experienced child marriage was associated with an increase of 0.102 in the child's *z*-score. In Model 2, the coefficient increased to 0.164(*p* < 0.05), suggesting a larger positive effect. Model 3 showed a further increase with a coefficient of 0.185 (*p* < 0.05). In Model 4, the coefficient is 0.126(*p* < 0.10), still indicating a positive effect, albeit at a lower significance level. Finally, Model 5 reported the highest coefficient at 0.202(*p* < 0.05), suggesting the strongest positive association. These consistent positive coefficients indicated that parental child marriage experience is robustly associated with better nutritional status in children. On the other hand, the interaction term between parental child marriage experience (yes/no) and parental food security status in 2007 is negative but not statistically significant across Models 2, 3, and 5, indicating no strong interaction effect.

Children's Food Consumption Score (FCS) and parental FCS in 2014 and 2007 were not statistically significant, suggesting they do not have a strong independent effect on children's nutritional status when other factors are controlled. In contrast, parent's BMI in 2014 was significant in Models 3, 4, and 5, indicating a positive association with children's z-scores; higher parental BMI is linked to better nutritional status in children. Neither children's nor parental food security status showed significant effects in the models they appear in.

The Table 3^7^ presents findings from binary logit regression models investigating the relationship between parental child marriage and children's likelihood of experiencing stunted growth, reported as odds ratios (OR) with corresponding standard errors (S.E.). In Model 1, children whose parents experienced child marriage had significantly higher odds of stunted growth (OR = 1.409, S.E. = 0.164, *p* < 0.001), highlighting a robust association between parental child marriage and child stunting. Model 2 introduced an interaction term between child marriage and parent food security in 2007, revealing a slightly increased odds ratio for child marriage (OR = 1.511, S.E. = 0.273, *p* < 0.01), though the interaction effect itself was not statistically significant (OR = 0.886, S.E. = 0.179).

Additionally, Model 3 demonstrated that child marriage is linked to higher odds of stunted growth (OR = 1.590, S.E. = 0.325, *p* < 0.01). Parental Food Consumption Score (FCS) in 2014 positively correlated with stunted growth (OR = 1.013, S.E. = 0.00368, *p* < 0.001), whereas FCS in 2007 exhibited a negative association (OR = 0.989, S.E. = 0.00528, *p* < 0.01). Model 4 confirmed the significance of child marriage with an odds ratio of 1.502 (S.E. = 0.327, *p* < 0.05). Parental FCS in 2014 remained positively significant (OR = 1.014, S.E. = 0.00379, *p* < 0.001), while parental BMI in 2014 showed a negative association (OR = 0.974, S.E. = 0.0147, *p* < 0.05). Furthermore, Model 5 included additional socio-economic factors and reintroduced the interaction term. Here, child marriage continued to exhibit an odds ratio of 1.377 (S.E. = 0.208, *p* < 0.01). Parental FCS in 2014 remained positively significant (OR = 1.014, S.E. = 0.00379, *p* < 0.001), and parental BMI in 2014 had a negative association (OR = 0.974, S.E. = 0.0147, *p* < 0.05). These findings collectively underscore the persistent association between parental child marriage and increased risk of children experiencing stunted growth across various model specifications.

### 3.3 Parental child marriage and children's food security

The Table 4^8^ presented findings from an OLS regression analysis examining the relationship between parental child marriage and children's FCS. The results highlighted several key determinants of children's food security. Parental child marriage experience had a strong negative association with children's FCS in Models 1 and 2, with coefficients of β* =* −18.35 (SE = 2.076) and β* =* −18.42 (SE = 2.071), respectively, both significant at the 1% level. This indicates that children whose parents experienced child marriage had significantly lower FCS. In Models 3 and 4, the negative association remained but was less pronounced.

**Table 4:** Parental child marriage and children's food consumption score—ordinary least squares regression.

	**Model 1**	**Model 2**	**Model 3**	**Model 4**
**Variables**	**Children's FCS**	**Children's FCS**	**Children's FCS**	**Children's FCS**
Parent's child marriage experience	-18.35^***^	-18.42^***^	-4.427^*^	-4.737^*^
	(2.076)	(2.071)	(2.590)	(2.724)
Interaction parental child marriage status^*^ parental FCS in 2007	0.340^***^	0.341^***^	0.0856^*^	0.110^**^
	(0.0355)	(0.0355)	(0.0450)	(0.0475)
Children's *z*-score		-0.000433	-0.000433	-4.04e-05
		(0.000483)	(0.000512)	(0.000549)
Children's malnutrition status		-1.136	-1.207	-2.214^**^
		(0.807)	(0.859)	(0.909)
Parent's BMI in 2014			0.128	0.0346
			(0.0806)	(0.0919)
Parent's BMI in 2007			-0.000199	-0.000913
			(0.00244)	(0.00194)
Socioeconomics characteristics	No	No	No	Yes
Interaction child marriage and parent food security in 2007 Constant	Yes	Yes	Yes	Yes
	60.57^***^	61.40^***^	41.22^***^	45.64^***^
	(0.411)	(0.725)	(2.720)	(8.860)
Observations	3,282	3,282	2,796	2,426
R-squared	0.031	0.032	0.086	0.117

Standard errors in parentheses.

* *p* < 0.05, ** *p* < 0.01, *** *p* < 0.001.

Robust standard errors are computed to account for heteroscedasticity or other forms of model misspecification. Model 4 incorporates socio-economic characteristics at various levels: individual (personal expenditure consumption, religion, ethnicity, education), household (household expenditure, expenditure shares for different food sources, and other factors), community (local laws on marriage, government policies on food and poverty eradication), and regional (regional GDP and Gini coefficient), and interaction variable.

The Table 4 also shows that the interaction effect between parental child marriage status and parental FCS in 2007 shows positive and significant coefficients across all models, suggesting that higher parental FCS in 2007 can mitigate the negative impact of parental child marriage on children's FCS. The coefficients were β* =* 0.340 (SE = 0.0355) and β* =* 0.341 (SE = 0.0355) in Models 1 and 2, significant at the 1% level. In Models 3 and 4, the coefficients were β* =* 0.0856 (SE = 0.0450) and β* =* 0.110 (SE = 0.0475), significant at the 10 and 5% levels, respectively.

Children's malnutrition status showed a negative but not statistically significant effect in Models 2 and 3. However, in Model 4, the coefficient is β* =* −2.214 (SE = 0.909), significant at the 5% level, indicating that malnutrition significantly reduces children's FCS. Moreover, parent's food security status consistently demonstrates a strong positive and statistically significant association in Models 3 (β* =* 3.158, SE = 0.411) and 4 (β* =* 3.848, SE = 0.725), both significant at the 1% level (see Table 4). This indicates that higher levels of parental food security significantly enhance children's FCS.

The Table 5^9^ presents findings from binary logit regression models that examined the relationship between parental child marriage and children's food security status, reported as odds ratios (OR) with corresponding standard errors (S.E.). In Model 1, children whose parents experienced child marriage had significantly lower odds of achieving food security status (OR = 0.151, S.E. = 0.0554, *p* < 0.001), indicating a strong negative association between parental child marriage and children's food security. Model 2 introduced an interaction term between child marriage and parent food security in 2007, showing a slight increase in the odds ratio for child marriage (OR = 1.037, S.E. = 0.00762, *p* < 0.001), while the interaction effect itself was also statistically significant (OR = 1.030, S.E. = 0.00926, *p* < 0.001). Furthermore, Model 3 indicates that child marriage remained negatively associated with children achieving food security (OR = 0.260, S.E. = 0.131, *p* < 0.001). In Model 4, children whose parents experienced child marriage had significantly lower odds of achieving food security status, with an odds ratio of 0.370 (S.E. = 0.214, *p* < 0.05). This finding underscores that children from families with a history of child marriage are notably less likely to attain food security compared to those without such a history. Furthermore, the interaction effect between child marriage and parent food security in 2007 yielded an odds ratio of 1.022 (S.E. = 0.0106, *p* < 0.05). This suggests that the combined impact of child marriage and parent food security in 2007 marginally enhances the likelihood of children achieving food security status, albeit with a modest effect.

**Table 5:** Parental child marriage and children's food consumption score–binary logit regression [odd ratio].

	**Model 1**	**Model 2**	**Model 3**	**Model 4**
**Variables**	**Food security status**	**Food security status**	**Food security status**	**Food security status**
Parent's child marriage status (Yes/No)	0.151^***^	0.210^***^	0.260^***^	0.370^*^
	(0.0554)	(0.0999)	(0.131)	(0.214)
Interaction child marriage and parent food security in 2007	1.037^***^	1.030^***^	1.025^***^	1.022^**^
	(0.00762)	(0.00926)	(0.00961)	(0.0106)
Child z score		1.000	1.000	1.000
		(0.000121)	(0.000132)	(0.000140)
Child's Malnutrition Status		0.669^**^	0.647^**^	0.603^***^
		(0.119)	(0.124)	(0.118)
Parent's BMI in 2014			0.986	0.970
			(0.0237)	(0.0272)
Parent's BMI in 2007			1.019	1.026
			(0.0183)	(0.0236)
Socioeconomics characteristics	No	No	No	Yes
Interaction child marriage and parent food security in 2007 Constant	Yes	Yes	Yes	Yes
	2.189^***^	2.547^***^	1.445^***^	-0.0829
	(0.0705)	(0.136)	(0.411)	(4.668)
Observations	3,282	3,282	2,796	2,426

Standard errors in parentheses.

* *p* < 0.05, ** *p* < 0.01, *** *p* < 0.001.

Robust standard errors are computed to account for heteroscedasticity or other forms of model misspecification. Model 4 incorporates socio-economic characteristics at various levels: individual (personal expenditure consumption, religion, ethnicity, education), household (household expenditure, expenditure shares for different food sources, and other factors), community (local laws on marriage, government policies on food and poverty eradication), regional (regional GDP and Gini coefficient), and interaction variables.

### 3.4 Additional analyzes: impact of gender, ethnicity, and religions

Table 6 depicts the relationship between child marriage status across genders, ethnicities, and religions, with outcomes related to children's likelihood of stunting (indicative of malnutrition) and their food security status. The log odds presented in Table 6 offer insights into how various factors relate to both stunting and food security status among children.

**Table 6:** Regression results on malnutrition and food security—heterogeneity.

	**Ethnicity—Ref: Javanese**		**Religion**		**Gender**	
**Variables**	**Stunting**	**FS**	**Stunting**	**FS**	**Stunting**	**FS**
Child marriage status	1.502^***^	0.159^***^	1.382^**^	0.152^***^	1.428^***^	0.157^***^
	(0.201)	(0.0583)	(0.181)	(0.0558)	(0.195)	(0.0581)
Sundanese	1.065	1.120				
	(0.136)	(0.210)				
Bali	1.047	1.009				
	(0.188)	(0.251)				
Bugis	1.255	0.671				
	(0.306)	(0.209)				
Chinese	0.534					
	(0.575)					
Maduranese	1.752^***^	1.304				
	(0.288)	(0.353)				
Sasak	2.618^***^	0.511^***^				
	(0.346)	(0.0868)				
Banjar	1.846^***^	1.557				
	(0.322)	(0.488)				
Bima Dompu	3.444^***^	1.883^*^				
	(0.617)	(0.703)				
Makassar	1.561	2.614				
	(0.484)	(-1.900)				
Palembang	6.559					
	(-7.631)					
Sumbawa	3.762^***^					
	(-1.442)					
Betawi	0.912	1.668				
	(0.336)	(0.975)				
Melayu	0.206	0.906				
	(0.209)	(0.712)				
Cirebon	1.024	2.489**				
	(0.219)	(-1.072)				
Interaction: Child marriage status * FS in 2007		1.037^***^		1.037^***^		1.037^***^
		(0.00756)		(0.00760)		(0.00763)
Islam			1.436^**^	0.872		
			(0.230)	(0.199)		
Female-headed household					1.189	1.537^*^
					(0.180)	(0.396)
Child's gender					0.831^**^	0.963
					(0.0653)	(0.112)
Parents' gender					0.944	0.864
					(0.0852)	(0.114)
Constant	0.259^***^	8.621^***^	0.245^***^	10.12^***^	0.484^***^	11.44^***^
	(0.0185)	(0.845)	(0.0381)	(-2.236)	(0.0860)	(-2.981)
Observations	3,266	3,266	3,282	3,282	3,282	3,282

Standard errors in parentheses.

* *p* < 0.05, ** *p* < 0.01, *** *p* < 0.001.

Robust standard errors are computed to account for heteroscedasticity or other forms of model misspecification. In this study, “Stunting” refers to the malnutrition status of children, assessed using height-for-age measurements to determine whether children are experiencing stunting. “FS” refers to the food security status of children. The analysis includes an interaction variable between child marriage and nutrition or food security status.

In this study, we aim to observe the differences across various ethnicities, rather than grouping them according to matriarchal or patriarchal systems. Indonesia's rich ethnic diversity is well-represented in the IFLS datasets, which include groups such as Javanese, Sundanese, Balinese, Batak, Bugis, Chinese, Maduranese, Sasak, Minang, Banjar, Bima Dompu, Makassar, Nias, Palembang, Sumbawa, Toraja, Betawi, Dayak, Melayu, Komering, Ambonese, Manado, Acehnese, other Southern Sumatrans, Banten, Cirebon, Gorontalo, and Kutai.

Children whose parents experienced child marriage had significantly higher odds of stunting (OR = 1.502, S.E. = 0.201, *p* < 0.001) and significantly lower odds of achieving food security (OR = 0.159, S.E. = 0.0583, *p* < 0.001). This indicates that child marriage is associated with an increased children's likelihood of being stunted and a decreased likelihood of food security among children. Moreover, the interaction term, analyzed across different demographic categories (ethnicity, religion, gender), consistently shows that the joint effect of child marriage and parent food security in 2007 slightly increases the odds of achieving food security (OR = 1.037, S.E. ranging from 0.00756 to 0.00763, *p* < 0.001). The Table 6 provides valuable insights into the likelihood of malnutrition status and FS status across different ethnicities. For malnutrition status, children who were married are 50.2% more likely to be malnourished, with this result being statistically significant at the 1% level (*p* < 0.01). While the Sundanese, Bali, Bugis, Chinese, and Betawi ethnicities exhibit varying degrees of higher or lower likelihood of malnutrition, these results are not statistically significant. In contrast, Maduranese individuals are 75.2% more likely, Sasak individuals are 161.8% more likely, and Banjar individuals are 84.6% more likely to be malnourished, with all these results being statistically significant at the 1% level.

Regarding FS status, children who were married have an 84.1% lower likelihood of having a positive FS status, also statistically significant at the 1% level. Sasak individuals are 48.9% less likely to have a positive FS status, significant at the 1% level. Other ethnic groups, such as the Sundanese, Bali, Bugis, Maduranese, Banjar, Makassar, Betawi, and Melayu, show non-significant variations in their likelihood of having a positive FS status. However, the data for Chinese, Palembang, and Sumbawa ethnicities regarding FS status is missing, so no interpretation can be made for these groups. Children from Islamic families have significantly higher odds of stunting (OR = 1.436, S.E. = 0.230, *p* < 0.01), although there is no significant effect on food security. Female-headed households exhibit significantly higher odds of achieving food security (OR = 1.537, S.E. = 0.396, *p* < 0.05), but there is no significant impact on stunting. Last but not least, female children have significantly lower odds of stunting (OR = 0.831, S.E. = 0.0653, *p* < 0.01).

## 4 Discussion

This study examines the impact of parental child marriage on food security and malnutrition in children under 15 years old. Parental socioeconomic status, food security, and nutritional status play a significant role in the malnutrition of their children. Parental feeding practices establish a social learning environment where children emulate the eating behaviors demonstrated by their parents (31). A study from (32) indicates that the primary predictors of stunting and underweight in children are food security and mothers' low body mass index.

Moreover, individuals who experience child marriage tend to have poorer physical, psychological, and reproductive health outcomes compared to their peers who marry at a later age (9). Women who marry at a young age and choose a husband of similar age are even less likely to be employed compared to other early-married women, although they are equally likely to work in the informal sector and earn similar hourly wages when they do (33). Unsurprisingly, their per capita household consumption is lower—by 8%—if they marry a younger man instead of an older one (33). These adverse effects can be passed across generations. Consequently, parents who experienced child marriage and subsequently encountered inadequate employment, limited economic resources, and insufficient education are likely to adopt suboptimal feeding practices, which in turn perpetuates the cycle of poverty and leads their children to adopt similar dietary habits. Thus, this aligns with the finding of (10), which highlight that Indonesian men and women who marry before turning 19 are likely to experience a lower quality of life.

This study reveals four main findings, namely: (1) the analysis consistently shows a significant and positive relationship between parental history of child marriage and children's BMI-for-age *z*-scores across all models, (2) to better understand the relationship between child marriage and nutritional outcomes, it is insufficient to only measure the association between BMI-for-age *z*-scores and parents' history of child marriage, (3) this study highlights a strong correlation between parental experience of child marriage and lower Food Consumption Scores (FCS) among children, (4) additional analysis (considering gender, religion, and ethnicity) revealed that child marriage significantly associates with the increased risk of children being stunted and reduced chances of achieving food security.

Firstly, the analysis consistently indicates a strong and positive association between parents' history of child marriage and children's BMI-for-age *z*-scores in all models tested which is relevant with the finding from Datta et al. (34), especially among women. Even though the higher *z*-score within a normal range indicates a healthier weight relative to BMI for age, however, a rise in *z*-scores to higher levels might suggest overweight or obesity. Obesity during childhood is associated with increased risk of premature death and adult disability. Furthermore, children who are overweight or obese are at greater risk of maintaining their weight status into adulthood. Moreover, Children born to child brides face a greater risk of malnutrition compared to those born to older mothers (35). This is consistent with the findings from (18), which indicate that child marriage is strongly associated with a higher risk of stunting and underweight in children. Additionally, they face an increased likelihood of developing noncommunicable diseases such as cardiovascular diseases, diabetes, musculoskeletal disorders, and cancers at younger ages (36).

Secondly, to gain deeper insight into the relationship between child marriage and nutritional outcomes, only measuring the association between BMI-for-age *z*-scores and parental history of child marriage is not enough. Therefore, we employed an additional analysis by examining child malnutrition status using height-for-age to better understand whether parent's child marriage experience related to children's likelihood of being stunted. We found that parental early marriage was associated with an increased likelihood of children experiencing stunted growth which can occur due to chronic nutritional deficiencies and related factors. Previous research by Paul et al. (18) similarly reported that child marriage (under 18 years) significantly increases the likelihood of stunting in children. Hence, this study's finding aligns with findings from Paul et al. (18) and complements the research by Wells et al. (37) and (38). Last but not least, a supporting finding from the prior study by Gambir et al. (39) highlights that girls who marry at a young age are more likely to suffer from malnutrition, and their children face an increased risk of malnutrition if their mothers were adolescents at the time of marriage.

Thirdly, this study underscores that parental experience of child marriage is strongly correlated with lower FCS among children. This negative association implies that children whose parents were involved in child marriage tend to experience reduced FCS, indicating poorer food security outcomes. This correlation can be attributed to the detrimental effects of child marriage, which are often associated with restricted agency, limited access to education and healthcare, and reduced empowerment (40). These factors can significantly impact food security and nutritional wellbeing. For instance, early marriage may disrupt a child's education, limiting their economic opportunities to access adequate food and acquire knowledge about healthy diets. This finding is supported by the prior study conducted by Cameron et al. (33), which demonstrates that women who marry early experience significantly lower life satisfaction and food consumption compared to those who marry later, as reflected in their subjective wellbeing index. Notably, the index for early-married women is nearly one full standard deviation lower than that of women who married at an older age.

Building on the previous explanation, the fourth finding of this study reveals that the association between child marriage and children's food security status yielded an odds ratio of 0.205, which corresponds to a reduction in the probability of ~17.05%. This underscores the potential detrimental impact of parental child marriage on children's food security outcomes, highlighting the critical need for targeted interventions to mitigate these effects and improve child wellbeing.

According to additional analysis (gender, religion, and ethnicity), child marriage emerges as a critical factor increasing the likelihood of children being stunted while decreasing their chances of food security. Specifically, children in households where child marriage occurred were more likely to experience stunting and face food insecurity, underscoring the adverse impact of early marriage on child development and nutrition. Moreover, ethnicity partly played a role, with certain ethnic groups showing higher odds of children being stunted, such as the Maduranese, Sasak, Banjar, and Bima-Dompu, while others exhibit lower odds of food security, including Bali, Bugis, and Islam. These variations highlight the importance of considering cultural contexts and specific ethnic disparities in addressing nutrition-related challenges among children. Furthermore, the household structure also influences outcomes, as evidenced by the slightly improved food security odds in female-headed households. This finding suggests potential differences in resource allocation or decision-making processes that may affect children's access to adequate nutrition.

In South Asia countries, finding from Subramanee et al. (41) highlights that both Hindu and Muslim religions are associated with the practice of child marriage. However, this association is inconclusive, as it varies significantly depending on the specific country and the prevailing religious majority within that region (41). In Indonesia, where the majority of the population is Muslim, child marriage is often justified by the absence of explicit Islamic teachings specifying a minimum marriage age (42). This justification is based on physical and biological maturity, specifically *aqil baligh* (coming of age) (42). Adherents refer to normative religious texts, claiming alignment with Islamic teachings. As a result, they believe that both women and men can marry once they achieve *aqil baligh*, regardless of their age (43). Furthermore, as noted by (44), pre-marital sex and the discovery of pregnancy before marriage—both considered violations of Islamic faith—are significant factors influencing the decision of girls, boys, or even their parents to proceed with child marriage. According to our result, in terms of religious determinants, children raised in Muslim households face higher log odds of stunting. This disparity may stem from cultural or dietary practices, socioeconomic factors, or differences in access to healthcare and education within Muslim communities. Importantly, our findings align with those of Banerjee (45), which also highlight an elevated risk of malnutrition among children in Muslim households. This observation may be influenced by various factors shaped by cultural and religious practices within Muslim households. For instance, dietary habits and food choices may vary based on religious beliefs and traditions, potentially leading to lower intake of calorie-dense or nutritionally rich foods. Practices like fasting during Ramadan could result in reduced calorie consumption among children during specific periods, affecting their overall nutritional status. Additionally, cultural norms related to body image and health perceptions may impact parental attitudes toward feeding practices, influencing children's dietary intake and subsequent growth patterns. Therefore, as a recommendation, more studies focusing on this issue would be valuable and insightful. Especially, according prior study from Subramanee et al. (41), which highlight that child marriage remains prevalent and is frequently concentrated in specific geographic areas or cultural groups, highlighting the need for more focused initiatives to safeguard adolescents from marriage. Such initiatives should take into account the underlying factors related to child marriage in the respective region (41).

Child marriage may not directly affect women's participation in the labor force, but the increased fertility linked with early marriage can impact their roles and the hours they can work (46). Regular breaks from employment due to childbirth and the substantial time commitment of caregiving responsibilities can also influence the types of employment women can pursue, often leading them to accept lower-paying and less secure jobs (46). These issues mirror the difficulties faced by women who marry during childhood (47).

The risk of child marriage is particularly high among girls living in rural areas, from impoverished communities, and with limited access to education (48). These girls often take on primary roles in leadership, decision-making, and social organization. Child marriage typically involves a minor, often a girl or boy, being wed to an adult, which constitutes a violation of their rights and wellbeing. This harmful practice is frequently linked to a lack of agency and limited access to education, healthcare, and empowerment (40), all of which can negatively affect their food security and nutritional status. Ultimately, child marriage is a violation of human rights that prevents girls from achieving their full potential (40).

Child marriage can diminish women's autonomy and restrict their ability to negotiate within their households, potentially including decisions about entering the workforce (46). The combined effect of lower socioeconomic status and reduced autonomy influences women's decisions regarding diet composition, physical activity, and health-seeking behaviors (49). Moreover, child marriage carries significant adverse effects (50), especially for women, who face health risks associated with early pregnancy and the cessation of education (51). Additionally, recent research suggests that child marriage among young men is more prevalent in rural areas and is associated with poverty and limited educational attainment (52).

The primary finding of our additional analyzes, especially in terms of gender dynamics, revealed that living in a female-headed household influences child nutrition outcomes. Children in female-headed households tend to have better food security. This is because the government prioritizes widows or female-headed households in their safety net programs, which helps families access a more diverse range of food. Moreover, mothers are more careful and thoughtful regarding their children's food. Additionally, the negative association with a child's gender indicates that girls in these households are less likely to experience stunting. These findings are consistent with those reported by Thurstans et al. (53).

Women aged 20–24 from low wellbeing households are significantly more likely to have married before the age of 18 (3). Specifically, 46.95% of these young brides lived in households that utilized food assistance programs (such as Raskin, Rastra, or BPNT), compared to only 38.71% of those who married at 18 or older (3). This disparity suggests a strong correlation between early marriage and economic hardship (3), with younger married women often coming from families that struggle financially. Consequently, living in low-wellbeing households can drive child marriage, perpetuating economic difficulties across generations. The existing social programs provided by the government have not effectively disrupted this cycle. While various international frameworks aim to eliminate child marriage, critics argue that many programs fail to engage with the underlying cultural beliefs that perpetuate the practice, particularly in the Indonesian context, where culture and ethnicity play a significant role. This calls into question the sustainability of such interventions and suggests the need for community-driven solutions that respect cultural sensitivities. Therefore, it is essential for the government to develop more targeted programs that consider the specific needs of different community groups, address the distinct needs of boys and girls, and emphasize better educational opportunities to empower the youth.

Raising community awareness about the economic, social, and emotional effects of child marriage is crucial for any successful initiative aimed at shifting cultural attitudes toward marriage (33). However, this endeavor presents significant challenges in Indonesia due to the diverse perspectives shaped by various religions and ethnicities, making it a sensitive issue. Therefore, it is important for the government to foster collaboration with other stakeholders to enhance the visibility of these efforts. By comprehending the implications of child marriage, individuals, families, and society as a whole can make more informed choices regarding marriage, ultimately benefiting from a shift away from this practice (33).

### 4.1 Limitations and strengths of the study

The strength of this study lies in its comparison of two distinct methodologies, OLS and Logit regression, which consistently yield robust results across analyzes, serving as a robustness check. The inclusion of diverse control variables effectively addresses potential confounding factors. Furthermore, heterogeneity analysis enriches the comprehensiveness of our findings, particularly relevant within the Indonesian context. Additionally, the utilization of unique longitudinal datasets adds novelty to this study. However, the study encounters several limitations. Firstly, the data is limited to two time points, 2007 and 2014. It exclusively examines parents and children residing in the same household, matching parents' individual IDs from 2007 with their information in 2014, and subsequently linking them with children under 15 years old using household IDs. Secondly, the analysis primarily relies on BMI-for-age z-scores, although other growth indicators were also considered for robustness checks. In the IFLS dataset, information on the timing of marriage is incomplete for some individuals, posing challenges in identifying instances of child marriage among parents. Consequently, we utilize the available data, specifically the year of marriage and year of birth, and include only those individuals for whom this information is available. For the dependent variable, we create a binary variable classifying individuals as either malnourished or of normal weight, without further disaggregating malnutrition into categories such as obese, overweight, or underweight. This suggests a potential avenue for future research to explore in greater detail. Additionally, it would be intriguing to utilize the most recent datasets available after 2014 from other Indonesian surveys.

### 4.2 Policy recommendation

Building on the insights gained from the study, several policy recommendations are pertinent for Indonesia and other developing countries facing similar challenges. Firstly, there should be targeted support for families affected by child marriage, which can include educational programs, economic assistance, and access to healthcare services to mitigate the long-term negative impacts on children's nutritional status. Secondly, public health interventions must be designed with cultural and religious sensitivity in mind. For instance, in Muslim communities, it is essential to ensure that nutritional programs take religious dietary practices into account while promoting balanced nutrition to combat both undernutrition and the risk of overweight.

Moreover, customizing programs to align with the unique characteristics of communities and family structures—such as ethnicity and religion—is crucial. This could involve implementing community-based initiatives that leverage these social structures to enhance child health outcomes. Additionally, it is important to adopt gender-sensitive approaches that recognize the differing nutritional needs and risks for boys and girls, ensuring that interventions effectively address malnutrition by considering these differences.

Last but not least, putting an end to child marriage can break the cycle of poverty across generations by empowering girls and women to participate more fully in society. When women and girls are educated and empowered, they can better provide essential nourishment and care for their children, leading to healthier and smaller families (48). Especially empowering girls to make better-informed choices about their futures, including aspects related to sex, reproduction, and marriage, as noted by Bandiera et al. (54).

## 5 Conclusion

From this study, we conclude that parental history of child marriage consistently shows a positive and significant association with children's *z*-scores. However, it is also positively correlated with children's likelihood of being stunted. Additionally, parental experience of child marriage is strongly negatively associated with children's FCS. Furthermore, this history correlates with a decreased probability of children being food secure.

Future policy recommendations include tailored interventions specifically designed for households with a history of child marriage. Furthermore, community-based programs that are tailored to the distinctive characteristics of different ethnicities, religious backgrounds, and family structures are essential for improving child health outcomes. Gender-sensitive policies are necessary to address the different nutritional needs and risks for boys and girls effectively. Lastly, ending child marriage is vital for breaking the cycle of poverty and improving the overall wellbeing of future generations, emphasizing the need for educational and empowerment initiatives for women and girls.

## Data Availability

The datasets presented in this study can be found in online repositories. The names of the repository/repositories and accession number(s) can be found at: https://www.rand.org/well-being/social-and-behavioral-policy/data/FLS/IFLS/access.html.

## Footnotes

^1^The model is adopted from Efevbera (11) and developed through extensive review of existing literature on girl child marriage, human development, and nutrition.

^2^See Appendix for detailed calculation method.

^3^Detailed further in Appendix.

^4^More information can be found in Appendix.

^5^This partial table is displayed here; the complete table can be accessed in the Appendix.

^6^The comprehensive table containing all control variables can be found in the Appendix.

^7^This partial table is displayed here; the complete table can be accessed in the Appendix.

^8^This partial table is displayed here; the complete table can be accessed in the Appendix.

^9^This partial table is displayed here; the complete table can be accessed in the Appendix.
