# Supplementary material for: The role of parental child marriage in children's food security and nutritional status: a prospective cohort study in Indonesia
Source: Front Public Health. 2024 Dec 10;12:1469483. doi: 10.3389/fpubh.2024.1469483 (PMC11666496; doi:10.3389/fpubh.2024.1469483)
Supplement: Supplementary file 1 [file Data_Sheet_1.zip › Supplementary Materials/Supplementary Material.pdf]

frontiers

Supplementary Material

0.1 More information

0.1.1 Covariates Variable

To address potential confounding factors (xt), we evaluated several key variables. Children's food security status (x1) is essential for understanding their nutritional well-being and its effect on household dynamics. Ensuring food security for children is critical as it directly impacts their physical health, cognitive development, and academic performance. Additionally, children's nutritional status can reflect the overall food availability and economic stability of the household. The interaction between parental child marriage status and parental food security status in 2007 (x2) helps explore the impact of early parental marriage on food security and related outcomes. Early marriage can disrupt educational and economic opportunities, affecting the household's ability to secure adequate food. Child gender and age (x3) are critical demographic variables that influence household resource allocation and dependency levels. Gender can affect the distribution of food and other resources, while age determines the nutritional and financial needs of household members. Child marriage status (yes/no) (x4) underscores the socio-economic implications of child marriage within households, as it often leads to higher dependency ratios and economic strain.

Parental food security status in 2007 and 2014 (x5) allows for an analysis of trends and changes in household stability and well-being over time, providing insights into long-term economic resilience or vulnerability. Parental malnutrition status in the same years is vital for understanding the health and nutritional status of parents and its effect on household expenditure, as poor parental health can increase healthcare costs and reduce economic productivity. Employment status (yes/no) (x6) serves as a direct indicator of income and economic stability within the household, influencing the ability to meet food and other essential needs.

Household size (x7) impacts the distribution of resources and overall expenditure, with larger households potentially facing greater economic strain. Religious affiliation (Islam, yes/no) can influence dietary practices, spending habits, and social norms, affecting household food security and expenditure patterns. Monthly household expenditure for non-food and food, along with the food expenditure share (x8), provides insight into the economic priorities and constraints of the household, highlighting how resources are allocated between essential and non-essential items. The age of household members (x9) affects dependency ratios and spending patterns, with younger and older members typically requiring more resources. Per capita monthly expenditure in 2007, adjusted to 2000 dollars (PPP) (x10), allows for a standardized comparison of economic well-being across different years, providing a clearer picture of economic trends. Moreover, we also added regional level information, namely: GDP in regional level and Gini index.

Rural residency (yes/no) (x11) influences access to resources, employment opportunities, and cost of living, with rural households often facing different economic challenges than urban ones. The gender of key household members (female, yes/no) impacts household decision-making and expenditure, as gender roles can affect economic activities and priorities. Marital status (married/cohabiting) (x12) affects household structure and economic support systems, influencing financial stability and

resource allocation. Belonging to a matriarchal ethnic group (yes/no) can influence social norms and household dynamics, affecting decision-making and economic behavior.

The availability of midwifery services in villages (x13) impacts family health and expenditures, as access to healthcare services can reduce maternal and infant mortality and related healthcare costs. The presence of ASKESKIN (health insurance for the poor) and RASKIN (subsidized rice programs) in villages (x14) affects healthcare and food security, respectively, providing safety nets for vulnerable households. Tracking rural residency in 2014 (yes/no) (x15) allows for the analysis of changes in access to resources and economic opportunities over time, highlighting shifts in rural development. Comparing per capita monthly expenditure in 2014 (PPP adjusted to 2000 dollars) to 2007 levels (x16) helps understand economic progress or decline, indicating the effectiveness of economic policies and programs. Finally, the enforcement of traditional laws and sanctions for breaking them, along with changes in traditional marriage laws (x17-x18), highlight the influence of cultural norms on household behavior and expenditure. By incorporating these variables, we aim to provide a thorough analysis of the factors influencing household expenditure and economic well-being.

0.1.2 To calculate FCS (Food Consumption Score)

To compute FCS, the following steps are employed, drawing on methodologies outlined in WFP (2015) and VAM (2015): 1) We extract data from both IFLS4 and IFLS5 regarding the frequency of food consumption within a week. Each food item is assigned a score based on consumption frequency, with a score of 1 indicating consumption once a week and a score of 2 indicating consumption twice a week, and so forth. 2) The consumption frequencies of all food items within the same food group are aggregated. If the sum of consumption frequencies for any food group exceeds 7, it is recoded as 7. 3) The aggregated consumption frequency for each food group is multiplied by its corresponding "importance weight" as specified in WFP (2015) and VAM (2015).

0.1.3 To Measure Child Nutritional Status

Compute the final z-score ing procedure is recommended to calculate a z-score for an indivi nt y at age t (WHO, 2007) :

$$\mathrm{z}^{*}_{\text{ind}} = \begin{cases} z_{\text{ind}} & \text{if } |z_{\text{ind}}| \leq 3 \\ 3 + \left(\frac{y-\text{SD3pos}}{\text{SD23pos}}\right) & \text{if } z_{\text{ind}} > 3 \\ -3 + \left(\frac{y-\text{SD3neg}}{\text{SD23neg}}\right) & \text{if } z_{\text{ind}} < -3 \end{cases}$$

where

•SD3pos is the cut-off 3 SD calculated at t by the LMS method:

$$\text{SD3pos} = M(t)\left[1 + L(t) \cdot S(t) \cdot (3)\right]^{\frac{1}{L(t)}};$$

•SD3neg is the cut-off -3 SD calculated at t by the LMS method:

$$\text{SD3neg} = M(t)\left[1 + L(t) \cdot S(t) \cdot (-3)\right]^{\frac{1}{L(t)}};$$

•SD23pos is the difference between the cut-offs 3 SD and 2 SD calculated at t by the LMS method:

$$\text{SD23pos} = M(t)\,[1 + L(t)\cdot S(t)\cdot(3)]^{\frac{1}{L(t)}} - M(t)\,[1 + L(t)\cdot S(t)\cdot(2)]^{\frac{1}{L(t)}}\,;$$

•SD23neg is the difference between the cut-offs -2 SD and -3 SD calculated at t by the LMS method:

$$\text{SD23neg} = M(t)\,[1 + L(t)\cdot S(t)\cdot(-2)]^{\frac{1}{L(t)}} - M(t)\,[1 + L(t)\cdot S(t)\cdot(-3)]^{\frac{1}{L(t)}}\,.$$

where

•SD3pos is the cut-off 3 SD calculated at t by the LMS method:

$$\text{SD3pos} = M(t)\,[1 + L(t)\cdot S(t)\cdot(3)]^{\frac{1}{L(t)}}\,;$$

•SD3neg is the cut-off -3 SD calculated at t by the LMS method:

$$\text{SD3neg} = M(t)\,[1 + L(t)\cdot S(t)\cdot(-3)]^{\frac{1}{L(t)}}\,;$$

•SD23pos is the difference between the cut-offs 3 SD and 2 SD calculated at t by the LMS method:

$$\text{SD23pos} = M(t)\,[1 + L(t)\cdot S(t)\cdot(3)]^{\frac{1}{L(t)}} - M(t)\,[1 + L(t)\cdot S(t)\cdot(2)]^{\frac{1}{L(t)}}\,;$$

•SD23neg is the difference between the cut-offs -2 SD and -3 SD calculated at t by the LMS method:

$$\text{SD23neg} = M(t)\,[1 + L(t)\cdot S(t)\cdot(-2)]^{\frac{1}{L(t)}} - M(t)\,[1 + L(t)\cdot S(t)\cdot(-3)]^{\frac{1}{L(t)}}\,.$$

0.2 Detailed Table Presented in Results

Table S1: Parental Child Marriage and Children's Z-Score - OLS

**Variables**	**Model (1)**	**Model (2)**	**Model (3)**	**Model (4)**	**Model (5)**
Parent's child marriage status (Yes/No)	0.102**	0.164**	0.185**	0.126*	0.202**
	(0.0470)	(0.0711)	(0.0931)	(0.0657)	(0.0939)
Interaction child marriage and parent food security in 2007		-0.0927	-0.152		-0.113
		(0.0786)	(0.112)		(0.0971)
Parent' FCS in 2014			-0.00161	-0.00107	-0.00108
			(0.00151)	(0.00155)	(0.00155)
Parent's FCS in 2007			0.00124	0.000836	0.00180
			(0.00285)	(0.00226)	(0.00239)
Parent's BMI in 2014			0.0101**	0.0158***	0.0153**
			(0.00503)	(0.00611)	(0.00618)
Parent's BMI in 2007			0.000154	0.000287**	0.000280**
			(0.000164)	(0.000134)	(0.000136)
Child's stunting status		0.0342	0.0438		
		(0.0472)	(0.0512)		
Parent's food security status			0.0497		
			(0.0985)		
Complete Junior High School				-0.0704	-0.0728
				(0.102)	(0.102)
Complete Senior High school				0.000172	-0.00156
				(0.0879)	(0.0880)
Incomplete elementary school				-0.0640	-0.0666
				(0.0675)	(0.0679)
Incomplete junior high school				-0.459**	-0.450**
				(0.203)	(0.202)
Incomplete senior high school				-0.350	-0.341
				(0.227)	(0.227)
No education				-0.184*	-0.189**

Table S1: Parental Child Marriage and Children's Z-Score - OLS

Variables	Model (1)	Model (2)	Model (3)	Model (4)	Model (5)
				(0.0953)	(0.0959)
Parent gender				-0.114*	-0.114*
				(0.0686)	(0.0685)
Separated				0.193	0.158
				(0.223)	(0.220)
Divorce				-0.134	-0.137
				(0.315)	(0.315)
Widow				0.282***	0.277***
Religion, ref: Islam				(0.0815)	(0.0813)
Protestand				-0.333	-0.336
				(0.314)	(0.314)
Chatolics				-0.136	-0.145
				(0.400)	(0.400)
Hinduism				-0.0585	-0.0620
				(0.147)	(0.147)
Parent's Personal					
Consumption Expenditure					
PCE in 2007				-6.24e-09	-6.25e-09
				(8.78e-09)	(8.76e-09)
GDP in 2007 (regional)				-1.09e-07	-1.12e-07
				(2.77e-07)	(2.77e-07)
Gini Index in 2007				-2.611	-2.547
				-2.289	-2.282
Household Expenditure in 2007				1.70e-08	1.70e-08
				(3.19e-08)	(3.20e-08)
Rice Expenditure Share in 2007				0.00101	0.00122
				(0.00839)	(0.00840)
Staple Expenditure					
Share in 2007 (except rice)				-0.00113	-0.00148

Table S1: Parental Child Marriage and Children's Z-Score - OLS

Variables	Model (1)	Model (2)	Model (3)	Model (4)	Model (5)
				(0.00884)	(0.00886)
Vegetables Expenditure Share in 2007				0.0138	0.0136
				(0.00844)	(0.00846)
Food Expenditure Share in 2007				0.00206	0.00219
				(0.00387)	(0.00388)
Oil Expenditure Share in 2007				-0.0114	-0.0112
				(0.0128)	(0.0128)
Medical Expenditure Share in 2007				0.0116**	0.0116**
				(0.00549)	(0.00550)
Clothes Expenditure Share in 2007				0.0116	0.0118
				(0.0140)	(0.0140)
Dairy Product Expenditure Share in 2007				-0.00413	-0.00435
				(0.00812)	(0.00815)
Education Expenditure Share in 2007				0.00183	0.00191
				(0.00268)	(0.00269)
House Expenditure Share in 2007				0.00124	0.00133
				(0.00323)	(0.00323)
Transfer Expenditure Share in 2007				-0.00888	-0.00858
				(0.00617)	(0.00614)
Alcohol Expenditure Share in 2007				0.00179	0.00172
Rice Expenditure Share in 2007				0.00101	0.00122
				(0.00839)	(0.00840)
Staple Expenditure Share in 2007 (except rice)				-0.00113	-0.00148
				(0.00884)	(0.00886)
Vegetables Expenditure Share in 2007				0.0138	0.0136
				(0.00844)	(0.00846)

Table S1: Parental Child Marriage and Children's Z-Score - OLS

Variables	Model (1)	Model (2)	Model (3)	Model (4)	Model (5)
Food Expenditure Share in 2007				0.00206	0.00219
				(0.00387)	(0.00388)
Oil Expenditure Share in 2007				-0.0114	-0.0112
				(0.0128)	(0.0128)
Medical Expenditure Share in 2007				0.0116**	0.0116**
				(0.00549)	(0.00550)
Clothes Expenditure Share in 2007				0.0116	0.0118
				(0.0140)	(0.0140)
Dairy Product Expenditure Share in 2007				-0.00413	-0.00435
				(0.00812)	(0.00815)
Education Expenditure Share in 2007				0.00183	0.00191
				(0.00268)	(0.00269)
House Expenditure Share in 2007				0.00124	0.00133
				(0.00323)	(0.00323)
Transfer Expenditure Share in 2007				-0.00888	-0.00858
				(0.00617)	(0.00614)
Alcohol Expenditure Share in 2007				0.00179	0.00172
Parent's Personal Consumption Expenditure in 2007				-6.24e-09	-6.25e-09
				(8.78e-09)	(8.76e-09)
GDP in 2007 (regional)				-1.09e-07	-1.12e-07
				(2.77e-07)	(2.77e-07)
Gini Index in 2007				-2.611	-2.547
				-2.289	-2.282
Household Expenditure in 2007				1.70e-08	1.70e-08
				(3.19e-08)	(3.20e-08)
Traditional Law of Enforcement in 2007				0.0119	0.0105
				(0.0630)	(0.0631)

Table S1: Parental Child Marriage and Children's Z-Score - OLS

**Variables**	**Model (1)**	**Model (2)**	**Model (3)**	**Model (4)**	**Model (5)**
Law broken Sanction in 2007				-0.0110	-0.0119
				(0.0782)	(0.0780)
The change of law in marriage				0.0899	0.0876
				(0.0673)	(0.0674)
Midwife services in village in 2007				0.0155	0.0133
				(0.344)	(0.344)
ASKESKIN program in village (2007)				0.0537	0.0557
				(0.167)	(0.167)
RASKIN program in village (2007)				-0.0488	-0.0509
				(0.347)	(0.346)
Market Operation program (2007)				-0.380**	-0.396**
				(0.182)	(0.183)
Log of regional GDP in 2014				-0.000802	0.000206
				(0.0528)	(0.0529)
Socioeconomics Characteristics	No	No	No	Yes	Yes
Interaction child marriage					
and parent food security in 2007	No	Yes	Yes	No	Yes
Constant	6.827***	6.885***	6.706***	6.278***	6.253***
	(0.0299)	(0.0805)	(0.163)	-1.303	-1.309
Observations	3,029	3,029	2,598	2,255	2,255
R-squared	0.003	0.004	0.009	0.044	0.045

Standard errors in parentheses

* $p < 0.05$, ** $p < 0.01$, *** $p < 0.001$

Note: Each observation in the dataset is weighted according to its significance in representing the target population. Robust standard errors are computed to account for heteroscedasticity or other forms of model misspecification. Models 4 and 5 incorporate socio-economic characteristics at various levels: individual (personal expenditure, religion, ethnicity, education),

household (expenditure shares for different food sources), community (local laws on marriage, government policies), and regional (regional GDP and Gini coefficient). Model 4 does not include an interaction variable, whereas Model 5 does.

Table S2: Parental Child Marriage and Children's Malnutrition Status - Logit Regression [Odd Ratio]

**Variables**	**Model (1)**	**Model (2)**	**Model (3)**	**Model (4)**	**Model (5)**
Parent's child marriage status (Yes/No)	1.409***	1.511**	1.590**	1.502*	1.377**
	(0.164)	(0.273)	(0.325)	(0.327)	(0.208)
Interaction child marriage and parent food security in 2007		0.886	0.931	0.881	
		(0.179)	(0.220)	(0.216)	
Parent food security status		1.001	1.000	1.002	1.002
		(0.00282)	(0.00323)	(0.00400)	(0.00399)
Child's food security status				0.959	0.965
				(0.244)	(0.245)
Parent' FCS in 2014			1.013***	1.014***	1.014***
			(0.00368)	(0.00379)	(0.00379)
Parent's FCS in 2007			0.989**	1.002	1.000
			(0.00528)	(0.00589)	(0.00530)
Parent's BMI in 2014			0.957***	0.974*	0.974*
			(0.0131)	(0.0147)	(0.0147)
Parent's BMI in 2007			1.001	1.000	1.000
			(0.000540)	(0.000493)	(0.000494)
Complete Junior High School				1.153	1.153
				(0.297)	(0.296)
Complete Senior High school				0.931	0.934
				(0.215)	(0.215)
Incomplete elementary school				1.263	1.265
				(0.216)	(0.216)
Incomplete junior high school				2.010**	1.991**
				(0.681)	(0.673)
Incomplete senior high school				2.055	2.039
				(0.926)	(0.915)
No education				1.350	1.356

Table S2: Parental Child Marriage and Children's Malnutrition Status - Logit Regression [Odd Ratio]

Variables	Model (1)	Model (2)	Model (3)	Model (4)	Model (5)
				(0.275)	(0.276)
Parent gender				1.140	1.140
				(0.170)	(0.170)
Separated				0.178	0.184
				(0.190)	(0.195)
Divorce				0.436	0.436
				(0.302)	(0.301)
Widow				0.639*	0.644*
Religion, ref: Islam				(0.167)	(0.168)
Protestand				0.580	0.583
				(0.469)	(0.470)
Chatolics				0.0598***	0.0599***
				(0.0639)	(0.0639)
Hinduism				0.638	0.637
				(0.188)	(0.188)
Regional GDPin 2007				1.000***	1.000***
				(3.39e-07)	(3.39e-07)
Regional Gini Index in 2007				0.0992	0.0935
				(0.476)	(0.447)
Household Expenditure in 2007				1.000**	1.000**
				(6.73e-08)	(6.74e-08)
Rice Expenditure Share in 2007				1.027*	1.027*
				(0.0159)	(0.0159)
Staple Expenditure Share in 2007 (except rice)				0.981	0.982
				(0.0162)	(0.0162)
Vegetables Expenditure Share in 2007				1.009	1.009
				(0.0232)	(0.0232)
Food Expenditure Share in 2007				1.012	1.012
				(0.00999)	(0.00998)

Table S2: Parental Child Marriage and Children's Malnutrition Status - Logit Regression [Odd Ratio]

Variables	Model (1)	Model (2)	Model (3)	Model (4)	Model (5)
Oil Expenditure Share in 2007				0.971	0.970
				(0.0330)	(0.0329)
Medical Expenditure Share in 2007				1.011	1.011
				(0.0157)	(0.0158)
Clothes Expenditure Share in 2007				1.003	1.003
				(0.0357)	(0.0357)
Dairy Product Expenditure Share in 2007				0.937***	0.937***
				(0.0223)	(0.0223)
Education Expenditure Share in 2007				0.998	0.998
				(0.00698)	(0.00701)
House Expenditure Share in 2007				0.995	0.995
				(0.00930)	(0.00930)
Transfer Expenditure Share in 2007				0.991	0.991
				(0.0140)	(0.0140)
Alcohol Expenditure Share in 2007				1.008	1.008
				(0.0132)	(0.0132)
Traditional Law of Enforcement in 2007				0.927	0.928
				(0.144)	(0.144)
Law broken Sanction in 2007				1.348*	1.350*
				(0.229)	(0.229)
The change of law in marriage				1.272*	1.275*
				(0.184)	(0.184)
Midwife services in village in 2007				1.108	1.106
				(0.936)	(0.934)
ASKESKIN program in village (2007)				0.188**	0.187**
				(0.157)	(0.156)
RASKIN program in village (2007)				3.552	3.578
				-3.919	-3.946
Market Operation program (2007)				1.006	1.025

Table S2: Parental Child Marriage and Children's Malnutrition Status - Logit Regression [Odd Ratio]

**Variables**	**Model (1)**	**Model (2)**	**Model (3)**	**Model (4)**	**Model (5)**
				(0.451)	(0.455)
Constant	0.300***	0.280***	0.604	0.418	0.443
	(0.0208)	(0.0519)	(0.247)	(0.739)	(0.778)
Observations	3,029	3,012	2,581	2,557	2,557

Standard errors in parentheses

* $p < 0.05$, ** $p < 0.01$, *** $p < 0.001$

Note: Each observation in the dataset is weighted according to its significance in representing the target population. Robust standard errors are computed to account for heteroscedasticity or other forms of model misspecification. Models 4 and 5 incorporate socio-economic characteristics at various levels: individual (personal expenditure, religion, ethnicity, education), household (expenditure shares for different food sources), community (local laws on marriage, government policies), and regional (regional GDP and Gini coefficient). Model 4 does not include an interaction variable, whereas Model 5 does.

Table S3: Parental Child Marriage and Children's Malnutrition Status - Logit Regression [Log Odds]

**Variables**	**Model (1)**	**Model (2)**	**Model (3)**	**Model (4)**	**Model (5)**
Parent's child marriage status (Yes/No)	0.343***	0.413**	0.464**	0.313**	0.406*
	(0.116)	(0.181)	(0.205)	(0.151)	(0.218)
Interaction child marriage and parent food security in 2007		-0.121	-0.0712		-0.136
		(0.202)	(0.236)		(0.246)
Child's FCS		0.00128	-0.000152	0.00229	0.00235
		(0.00282)	(0.00323)	(0.00401)	(0.00402)
Child food security status				-0.0348	-0.0416
				(0.256)	(0.257)
Parent' FCS in 2014			0.0125***	0.0141***	0.0141***
			(0.00363)	(0.00378)	(0.00378)
Parent's FCS in 2007			-0.0110**	0.000706	0.00199
			(0.00534)	(0.00536)	(0.00594)
Parent's BMI in 2014			-0.0436***	-0.0273*	-0.0279*
			(0.0137)	(0.0154)	(0.0154)
Parent's BMI in 2007			0.000597	0.000261	0.000253
			(0.000539)	(0.000504)	(0.000502)
Complete Junior High School				0.135	0.135
				(0.258)	(0.258)
Complete Senior High school				-0.107	-0.109
				(0.233)	(0.233)
Incomplete elementary school				0.233	0.231
				(0.172)	(0.173)
Incomplete junior high school				0.632*	0.643*
				(0.347)	(0.348)
Incomplete senior high school				0.698	0.706
				(0.454)	(0.456)
No education				0.278	0.273

Table S3: Parental Child Marriage and Children's Malnutrition Status - Logit Regression [Log Odds]

Variables	Model (1)	Model (2)	Model (3)	Model (4)	Model (5)
				(0.207)	(0.207)
Parent gender				0.141	0.141
				(0.150)	(0.150)
Separated				-1.689	-1.722
				-1.067	-1.071
Divorce				-0.829	-0.827
				(0.696)	(0.696)
Widow				-0.479*	-0.486*
Religion, ref: Islam				(0.268)	(0.269)
Protestand				-1.025	-1.029
				-1.094	-1.094
Chatolics				-2.798***	-2.799***
				-1.070	-1.070
Hinduism				-0.578*	-0.577*
				(0.322)	(0.322)
PCE in 2007				-3.03e-09	-3.02e-09
				(3.48e-08)	(3.49e-08)
Regional GDPin 2007				-1.86e-06***	-1.87e-06***
				(7.14e-07)	(7.12e-07)
Regional Gini Index in 2007				-1.616	-1.515
				-5.249	-5.265
Household Expenditure in 2007				-1.57e-07	-1.57e-07
				(1.01e-07)	(1.02e-07)
Rice Expenditure Share in 2007				0.0281*	0.0285*
				(0.0162)	(0.0161)
Staple Expenditure Share in 2007 (except rice)				-0.0193	-0.0198
				(0.0166)	(0.0166)
Vegetables Expenditure Share in 2007				0.00735	0.00723
				(0.0231)	(0.0231)

Table S3: Parental Child Marriage and Children's Malnutrition Status - Logit Regression [Log Odds]

Variables	Model (1)	Model (2)	Model (3)	Model (4)	Model (5)
Food Expenditure Share in 2007				0.0118	0.0120
				(0.00975)	(0.00976)
Oil Expenditure Share in 2007				-0.0318	-0.0310
				(0.0343)	(0.0343)
Medical Expenditure Share in 2007				0.0112	0.0112
				(0.0158)	(0.0157)
Clothes Expenditure Share in 2007				0.000181	0.000582
				(0.0371)	(0.0371)
Dairy Product Expenditure Share in 2007				-0.0657***	-0.0663***
				(0.0236)	(0.0237)
Education Expenditure Share in 2007				-0.000866	-0.000715
				(0.00709)	(0.00706)
House Expenditure Share in 2007				-0.00523	-0.00520
				(0.00942)	(0.00942)
Transfer Expenditure Share in 2007				-0.00813	-0.00774
				(0.0141)	(0.0141)
Alcohol Expenditure Share in 2007				0.00772	0.00757
				(0.0131)	(0.0131)
Traditional Law of Enforcement in 2007				-0.0532	-0.0554
				(0.158)	(0.158)
Law broken Sanction in 2007				0.296*	0.294*
				(0.172)	(0.172)
The change of law in marriage				0.243	0.240
				(0.151)	(0.151)
Midwife services in village in 2007				0.106	0.108
				(0.845)	(0.846)
ASKESKIN program in village (2007)				-1.652**	-1.644**
				(0.808)	(0.808)
RASKIN program in village (2007)				1.265	1.257

Table S3: Parental Child Marriage and Children's Malnutrition Status - Logit Regression [Log Odds]

**Variables**	**Model (1)**	**Model (2)**	**Model (3)**	**Model (4)**	**Model (5)**
				-1.085	-1.085
Market Operation program (2007)				0.0460	0.0268
				(0.431)	(0.435)
Log of GDP Regional in 2014				0.101	0.104
				(0.139)	(0.138)
Socioeconomics Characteristics	No	No	No	Yes	Yes
Interaction child marriage and parent food security in 2007	No	Yes	Yes	No	Yes
Constant	-1.205***	-1.272***	-0.505	-2.109	-2.215
	(0.0693)	(0.185)	(0.410)	-2.655	-2.661
Observations	3,029	3,012	2,581	2,518	2,518

Standard errors in parentheses

* $p < 0.05$, ** $p < 0.01$, *** $p < 0.001$

Note: Each observation in the dataset is weighted according to its significance in representing the target population. Robust standard errors are computed to account for heteroscedasticity or other forms of model misspecification. Models 4 and 5 incorporate socio-economic characteristics at various levels: individual (personal expenditure, religion, ethnicity, education), household (expenditure shares for different food sources), community (local laws on marriage, government policies), and regional (regional GDP and Gini coefficient). Model 4 does not include an interaction variable, whereas Model 5 does.

Table S4: Parental Child Marriage and Children's Food Security Status - Logit Regression [Odd Ratio]

**Variables**	**Model (1)**	**Model (2)**	**Model (3)**	**Model (4)**
Parent's child marriage status (Yes/No)	0.151***	0.210***	0.260***	0.370*
	(0.0554)	(0.0999)	(0.131)	(0.214)
Interaction child marriage and parent food security in 2007	1.037***	1.030***	1.025***	1.022**
	(0.00762)	(0.00926)	(0.00961)	(0.0106)
Child z score		1.000	1.000	1.000
		(0.000121)	(0.000132)	(0.000140)
Child's Malnutrition Status		0.669**	0.647**	0.603***
		(0.119)	(0.124)	(0.118)
Parent's BMI in 2014			0.986	0.970
			(0.0237)	(0.0272)
Parent's BMI in 2007			1.019	1.026
			(0.0183)	(0.0236)
food_secure_parent			1.540**	1.102
			(0.279)	(0.230)
Complete Junior High School				1.217
				(0.422)
Complete Senior High school				2.389**
				(0.960)
Incomplete elementary school				0.953
				(0.222)
Incomplete junior high school				1.813
				-1.061
Incomplete senior high school				3.912
				-3.822
No education				0.903
				(0.239)
Parent gender				0.820
				(0.193)

Table S4: Parental Child Marriage and Children's Food Security Status - Logit Regression

Variables	Model (1)	Model (2)	Model (3)	Model (4)
Separated				3.847
				-4.549
Divorce				1.322
				(0.878)
Widow				1.626
Religion, ref: Islam				(0.591)
Protestand				0.447
				(0.381)
Chatolics				1.059
				-1.529
Hinduism				1.011
				(0.495)
Regional GDPin 2007				1.000
				(1.07e-06)
Regional Gini Index in 2007				1,95
				-17,629
Regional Gini Index in 2014				160.5
				(607.0)
Household Expenditure in 2007				1.000
				(1.67e-07)
Rice Expenditure Share in 2007				1.008
				(0.0261)
Staple Expenditure Share in 2007 (except rice)				1.041
				(0.0266)
Vegetables Expenditure Share in 2007				1.046
				(0.0322)
Food Expenditure Share in 2007				0.968***
				(0.0119)
Oil Expenditure Share in 2007				1.032

Table S4: Parental Child Marriage and Children's Food Security Status - Logit Regression

**Variables**	**Model (1)**	**Model (2)**	**Model (3)**	**Model (4)**
				(0.0446)
Medical Expenditure Share in 2007				1.008
				(0.0216)
Clothes Expenditure Share in 2007				1.012
				(0.0526)
Dairy Product Expenditure Share in 2007				1.105***
				(0.0361)
Education Expenditure Share in 2007				0.988
				(0.00857)
House Expenditure Share in 2007				0.963***
				(0.0105)
Transfer Expenditure Share in 2007				1.054***
				(0.0215)
Alcohol Expenditure Share in 2007				1.053***
				(0.0180)
Traditional Law of Enforcement in 2007				1.058
				(0.233)
Law broken Sanction in 2007				0.655*
				(0.167)
The change of law in marriage				0.802
				(0.167)
Midwife services in village in 2007				3.089
				-2.268
ASKESKIN program in village (2007)				0.836
				(0.666)
RASKIN program in village (2007)				0.789
				(0.821)
Market Operation program (2007)				2.774
				-2.092

Table S4: Parental Child Marriage and Children's Food Security Status - Logit Regression

**Variables**	**Model (1)**	**Model (2)**	**Model (3)**	**Model (4)**
Log of Regional GDPin 2014				0.851
				(0.195)
Constant	8.929***	12.62***	8.153***	0.384
	(0.629)	-2.320	-4.348	-1.609

Standard errors in parentheses

* $p < 0.05$, ** $p < 0.01$, *** $p < 0.001$

Note: Each observation in the dataset is weighted according to its significance in representing the target population. Robust standard errors are computed to account for heteroscedasticity or other forms of model misspecification. Models 4 and 5 incorporate socio-economic characteristics at various levels: individual (personal expenditure, religion, ethnicity, education), household (expenditure shares for different food sources), community (local laws on marriage, government policies), and regional (regional GDP and Gini coefficient). Model 4 does not include an interaction variable, whereas Model 5 does.

Table S5: Parental Child Marriage and Children's Food Security Status - Logit Regression

**Variables**	**Model (1)**	**Model (2)**	**Model (3)**	**Model (4)**
Parent's child marriage status (Yes/No)	-1.892***	-1.934***	-1.759***	-1.591***
	(0.368)	(0.372)	(0.396)	(0.464)
Interaction child marriage and parent FCS in 2007	0.0366***	0.0374***	0.0321***	0.0331***
	(0.00734)	(0.00745)	(0.00783)	(0.00932)
Child z score		-0.000233***	-0.000151	-0.000111
		(8.77e-05)	(9.83e-05)	(0.000108)
Child's Malnutrition Status		-0.203	-0.298**	-0.376**
		(0.132)	(0.144)	(0.157)
Parent's BMI in 2014			0.00711	0.00146
			(0.0186)	(0.0252)
Parent's BMI in 2007			0.0217	0.0272
			(0.0155)	(0.0223)
Parent food security status in 2014			0.632***	0.457***
			(0.135)	(0.158)
Complete Junior High School				0.127
				(0.276)
Complete Senior High school				0.638**
				(0.283)
Incomplete elementary school				-0.0580
				(0.177)
Incomplete junior high school				0.963*
				(0.525)
Incomplete senior high school				1.584
				-1.053
No education				0.00202
				(0.233)
Parent gender				-0.139
				(0.179)

Table S5: Parental Child Marriage and Children's Food Security Status - Logit Regression

Variables	Model (1)	Model (2)	Model (3)	Model (4)
Separated				0.639
				-1.084
Divorce				0.194
				(0.508)
Widow				0.0526
Religion, ref: Islam				(0.297)
Protestand				-0.351
				(0.809)
Chatolics				0.864
				-1.171
Hinduism				-0.0124
				(0.405)
Parent's Personal Consumption Expenditure in 2007				9.21e-08*
				(5.09e-08)
Log of regional GDP in 2007				6.22e-07
				(8.52e-07)
Regional Gini Index in 2007				9.694
				-7.026
Household Expenditure in 2007				-8.45e-08
				(1.03e-07)
Rice Expenditure Share in 2007				-0.0629**
				(0.0318)
Staple Expenditure Share in 2007 (except rice)				0.0939***
				(0.0323)
Vegetables Expenditure Share in 2007				0.0306
				(0.0258)
Food Expenditure Share in 2007				-0.0265***

Table S5: Parental Child Marriage and Children's Food Security Status - Logit Regression

Variables	Model (1)	Model (2)	Model (3)	Model (4)
				(0.00990)
Oil Expenditure Share in 2007				0.0807**
				(0.0378)
Medical Expenditure Share in 2007				-0.0268
				(0.0173)
Clothes Expenditure Share in 2007				-0.0198
				(0.0419)
Dairy Product Expenditure Share in 2007				0.0720***
				(0.0261)
Education Expenditure Share in 2007				-0.00937
				(0.00791)
House Expenditure Share in 2007				-0.0249***
				(0.00905)
Transfer Expenditure Share in 2007				0.0369**
				(0.0173)
Alcohol Expenditure Share in 2007				0.0178
				(0.0128)
Traditional Law of Enforcement in 2007				-0.170
				(0.179)
Law broken Sanction in 2007				-0.0596
				(0.212)
The change of law in marriage				-0.0796
				(0.163)
Midwife services in village in 2007				0.230
				(0.689)
Health care card in village (2007)				-0.668
				(0.486)
ASKESKIN program in village (2007)				-0.822
				(0.901)

Table S5: Parental Child Marriage and Children's Food Security Status - Logit Regression

Variables	Model (1)	Model (2)	Model (3)	Model (4)
RASKIN program in village (2007)				0.619
				-1.158
Market Operation program (2007)				1.153*
				(0.651)
Log of PCE in 2014				-0.0729
				(0.0897)
Log of Regional GDP in 2014				-0.0884
				(0.170)
Log of Gini in 2014				-0.484
				-1.394
Socioeconomics characteristics	No	No	No	Yes
Constant	2.189***	2.547***	1.445***	-0.0829
	(0.0705)	(0.136)	(0.411)	-4.668
Observations	3,282	3,282	2,796	2,426

Robust standard errors in parentheses
*** p<0.01, ** p<0.05, * p<0.1

Table S6: Parental Child Marriage and Children's FCS-OLS

Variables	Model (1)	Model (2)	Model (3)	Model (4)
Parent's child marriage status (Yes/No)	-18.35***	-18.42***	-4.427*	-4.737*
	-2.076	-2.071	-2.590	-2.724
Child z score		-0.000433	-0.000433	-4.04e-05
		(0.000483)	(0.000512)	(0.000549)
Parent FCS in 2014			0.237***	0.221***
			(0.0271)	(0.0291)
Parent FCS in 2007			0.0503	-0.0361
			(0.0380)	(0.0421)
Parent's BMI in 2014			0.128	0.0346
			(0.0806)	(0.0919)
Parent's BMI in 2007			-0.000199	-0.000913
			(0.00244)	(0.00194)
Malnutrition status child		-1.136	-1.207	-2.214**
		(0.807)	(0.859)	(0.909)
Parent food security 2014			3.158***	3.848***
			-1.120	-1.190
Complete Junior High School				2.485*
				-1.484
Complete Senior High school				3.541***
				-1.258
Incomplete elementary school				-0.203
				-1.003
Incomplete junior high school				-0.0457
				-2.115
Incomplete senior high school				5.483**
				-2.724
No education				0.612
				-1.371
PCE in 2007				7.19e-07***

Table S6: Parental Child Marriage and Children's FCS - OLS

Variables	Model (1)	Model (2)	Model (3)	Model (4)
				(1.43e-07)
Household Expenditure in 2007				-7.42e-07*
				(4.18e-07)
Food Expenditure Share in 2007				0.0371
				(0.0299)
Alcohol Expenditure Share in 2007				-0.0218
				(0.0635)
Traditional Law of Enforcement in 2007				1.089
				(0.889)
Law broken Sanction in 2007				-2.758***
				-1.020
The change of law in marriage				0.593
				(0.870)
Midwife services in village in 2007				-1.611
				-3.688
Health care card in village (2007)				-5.214**
				-2.320
ASKESKIN program in village (2007)				-1.209
				-3.373
RASKIN program in village (2007)				4.673
				-4.791
Log PCE 2014				-0.352
				(0.474)
Interaction child marriage and parent FCS in 2007	0.340***	0.341***	0.0856*	0.110**
	(0.0355)	(0.0355)	(0.0450)	(0.0475)
Socioeconomics characteristics	NO	NO	NO	YES
Constant	60.57***	61.40***	41.22***	45.64***
	(0.411)	(0.725)	-2.720	-8.860

Table S6: Parental Child Marriage and Children's FCS - OLS

**Variables**	**Model (1)**	**Model (2)**	**Model (3)**	**Model (4)**
Observations	3,282	3,282	2,796	2,426
R-squared	0.031	0.032	0.086	0.117

0.3 Additional Table

Table S7: Children's Nutritional Status

Growth Indicator	Malnutrition	urban +rural (%)	Urban(%)	Rural(%)
Weight for Age	NA	66.24	65.96	66.55
	normal	25.32	25.32	25.31
	see note 2	2.36	2.57	2.12
	severely underweight	1.28	1.54	1.00
	underweight	4.81	4.61	5.03
Height for Age	NA	0.70	0.61	0.80
	normal	72.74	72.75	72.73
	see note 1	0.26	0.18	0.36
	severely stunted	6.96	7.71	6.11
	stunted	19.34	18.75	20.00
Weight for Height	NA	45.47	45.86	45.03
	normal	44.75	44.14	45.43
	obese	2.22	2.43	2.00
	overweight	2.13	2.29	1.96
	possible risk of overweight, see note 3	3.71	3.54	3.91
	severely wasted	0.26	0.29	0.24
	wasted	1.45	1.46	1.44
BMI for Age	NA	0.81	0.79	0.84
	normal	75.55	75.57	75.53
	obese	2.38	2.61	2.12
	overweight	4.37	4.39	4.35
	possible risk of overweight, see note 3	7.90	8.00	7.78
	severely wasted	2.47	2.75	2.16
	wasted	6.52	5.89	7.23
